# Radiographic and Histomorphometric Evaluation of Biomaterials Used for Lateral Sinus Augmentation: A Systematic Review on the Effect of Residual Bone Height and Vertical Graft Size on New Bone Formation and Graft Shrinkage

**DOI:** 10.3390/jcm10214996

**Published:** 2021-10-27

**Authors:** Paolo Pesce, Maria Menini, Luigi Canullo, Shahnawaz Khijmatgar, Laura Modenese, Gianmarco Gallifante, Massimo Del Fabbro

**Affiliations:** 1 Department of Surgical Sciences (DISC), University of Genoa, Ospedale S. Martino, L. Rosanna Benzi 10, 16132 Genoa, Italy; maria.menini@unige.it (M.M.); modenese.laura@gmail.com (L.M.); gmarco.gallifante@gmail.com (G.G.); 2 Department of Periodontology, University of Bern, 3012 Bern, Switzerland; luigicanullo@yahoo.com; 3 Department of Biomedical, Surgical, and Dental Sciences, University of Milan, 20122 Milan, Italy; khijmatgar@gmail.com (S.K.); massimo.delfabbro@unimi.it (M.D.F.); 4 IRCCS Orthopedic Institute Galeazzi, 20161 Milan, Italy

**Keywords:** sinus lift, bone regeneration, biomaterials, grafts

## Abstract

The aim of the present systematic review was to investigate the effect of residual bone height (RBH) and vertical bone gain on new bone formation (NBF) and graft shrinkage after lateral sinus lifts using different biomaterials. Methods: An electronic search was conducted on three databases to identify randomized controlled trials (RCTs) published until January 2021 with at least one follow-up at 6 months and at least five patients treated, comparing biomaterials used for maxillary sinus augmentation with a lateral approach. Graft volumetric changes, RBH, vertical bone gain, implant failure, and post-operative complications were evaluated. The risk of bias was assessed using the Cochrane tool. Results: We used 4010 identified studies, of which 21 were RCTs. Overall, 412 patients and 533 sinuses were evaluated. Only three publications had an overall low risk of bias. After 6 months, xenograft (XG) showed the least volume reduction (7.30 ± 15.49%), while autogenous graft (AU) was the most reabsorbed (41.71 ± 12.63%). NBF appeared to not be directly correlated with RBH; on the contrary, the overall linear regression analysis showed that NBF significantly decreased by 1.6% for each mm of postoperative vertical graft gain. This finding suggests that the greater the augmentation, the lower the NBF. A similar tendency, with a regression coefficient even higher than the overall one, was also observed with alloplast (AP) and XG. Conclusions: The present results suggested that NBF was essentially independent of preoperative bone height. On the contrary, the smaller the volume was of the graft placed, the higher the amount of new bone formed, and the smaller the graft shrinkage was. Minimizing the augmentation volume might be beneficial to graft healing and stability especially when using AP and XG.

## 1. Introduction

In the posterior maxilla, where the possible lack and/or low quality of bone associated with the pneumatisation of the sinus represent a risk for implant osseointegration [1,2,3], the implant rehabilitation is often combined with regenerative procedures such as a lateral or transcrestal maxillary sinus augmentation [4,5,6,7].

In 1978, Tatum was the first to practise bone grafting under the sinus membrane [8], and in 1980, Boyne and James performed the first lateral maxillary sinus augmentation using the Caldwell–Luc technique [9]. 

Even if biological complications occur with this technique [10,11], standard length implants can be used, as opposed to short implants, to increase the bone-implant interface, improve primary stability, and reduce implant failure [12,13]. Although alternative rehabilitation strategies have been proposed such as the use of tilted implants [14,15], the successful combination of sinus augmentation and standard implant placement has been strongly supported in the scientific literature. In 1996, Jensen et al. reported a success rate of 90% three years post-loading [16]. Similar or better results have been reported by several subsequent studies and systematic reviews [17,18,19,20]. 

As an alternative to autogenous bone graft (AB), several biomaterials such as allograft (AG), xenograft (XG), and alloplastic material (AP), either alone or in combination, have been proposed as grafting materials for sinus lifts [21]. While osteoconductive properties characterize AG, XG, and AP, autogenous bone is the only graft material presenting an osteoinductive and osteogenic capacity [22]. For these reasons, the use of AB is still considered the gold standard even though it can lead to post-operative complications at the donor site and is associated with greater morbility, limited availability, and significant resorption [23]. While the use of only AB in maxillary sinus lifts guarantees a high implant survival rate [16,18], the autograft is often mixed with slowly resorbing materials such as XG [24]. 

XG has demonstrated its effectiveness in maxillary sinus floor augmentation, both alone and combined with AB [25,26,27]. XGs are good space-makers and very slowly resorbable biomaterials. Histological studies have reported the presence of intact xenograft particles, 3–11 years post-sinus lift (SL), showing no significant dimensional changes, as compared to those observed six months after surgery [28,29].

Alloplastic materials such as polymers, calcium sulphates, hydroxyapatite (HA), calcium phosphates, and bioactive glass are inorganic osteoconductive bone substitutes [23,24]. An histomorphometric study comparing an AG (composed of freeze-dried bone allograft (FDBA)) and an AP (composed of biphasic calcium phosphate (BCP)) proved that the use of an AG determines similar new bone formation and a reduced amount of residual graft with respect to AP, concluding that the latter is less osteoconductive [30].

Demineralized, freeze-dried bone allograft (DFDBA) and FDBA are space-occupying osteoconductive biomaterials that have only slight osteoinductive properties [31,32]. In addition, various samples of DFDBA from different bone banks may have dissimilar potentiality to promote new bone formation, depending on the donor’s age, as demonstrated by Schwartz et al. [33].

Several recent reviews based on histomorphometric data comparing different graft materials used in SL agree on the supremacy of AB to induce new bone formation [34,35,36], as opposed to other authors such as Trimmel et al. who have supported the use of a composite graft made of XG and bone marrow concentrate (BMC) [37].

The aim of the present review was therefore to analyse the effects of residual bone height (RBH) and vertical bone gain on the new bone formation (NBF) and the graft shrinkage after lateral sinus lifts using different biomaterials. A second aim was to test if the dimensional baseline variables such as the residual bone height and the initial size of the graft may play a role on the shrinkage and the new bone formation.

## 2. Materials and Methods

### 2.1. Protocol and Registration

The present systematic review followed the guidelines of transparent reporting of systematic reviews and meta-analyses (PRISMA), and the protocol was registered on Prospero with the code CRD42020199261.

The following focused questions were analysed in accordance with the PICO strategy:

1. What was the effect of residual bone height and post-operative vertical change on new bone formation in sinus augmentation procedures 6 months after loading? 

Population: Healthy patients with an atrophic posterior maxilla requiring lateral sinus augmentation;

Intervention: Sinus augmentation with any biomaterial;

Comparison: Any comparison between materials included in the intervention or with augmentation without adding biomaterials;

Outcomes: Review of new bone formation, implant survival, and complications.

2. What was the shrinkage of different bone substitutes when used in sinus augmentation procedures 6 months after surgery?

Population: Healthy patients with an atrophic posterior maxilla requiring lateral sinus augmentation;

Intervention: Sinus augmentation with any biomaterial;

Comparison: Any comparison between materials included in the intervention or with augmentation without adding biomaterials;

Outcomes: Graft volumetric changes measured with CBCT, implant survival, and complications.

### 2.2. Search Strategy

The research of the articles was performed online using three main databases: PubMed/MEDLINE, Scopus, and the Cochrane Central Register of Controlled Clinical Trials (CENTRAL). The investigation began in April 2020 and ended in January 2021. The survey did not exclude any article by year of publication or language and was performed using the following search strategy: “(maxillary sinus lift OR maxillary sinus augmentation OR maxillary sinus elevation OR maxillary sinus grafting OR maxillary sinus surgery OR sinus graft) AND dental implants” that was modified for each database. A by-hand search was also performed in the following journals: the *British Dental Journal*, the *British Journal of Oral and Maxillofacial Surgery*, *Clinical Implant Dentistry and Related Research*, *Clinical Oral Implants Research*, *Clinical Oral Investigations*, the *European Journal of Oral Sciences*, *Implant Dentistry*, the *International Journal of Oral and Maxillofacial Implants*, the *International Journal of Oral and Maxillofacial Surgery*, the *International Journal of Oral Implantology* (formerly, the *European Journal of Oral Implantology*), the *International Journal of Periodontics and Restorative Dentistry*, the *Journal of Clinical Periodontology*, the *Journal of Dental Research*, the *Journal of Dentistry*, the *Journal of Maxillofacial and Oral Surgery*, the *Journal of Oral and Maxillofacial Surgery*, the *Journal of Periodontal Research*, and the *Journal of Periodontology*.

The reference lists of all identified RCTs and relevant systematic reviews were scanned for any additional studies. Online registries providing information about in-progress clinical trials and the grey literature were checked (http://clinicaltrials.gov/; http://www.centerwatch.com/clinicaltrials/; http://www.clinicalconnection.com/; www.greylit.org; www.opengrey.eu; accessed on 1 October 2021).

### 2.3. Eligibility Criteria

Only randomized controlled trials (RCTs), both split-mouth and parallel, that included a test and a control group or two or more comparisons were included. Only studies in English with at least one post-surgery follow-up at 6 months and at least 5 patients treated were included. Non-randomized clinical studies, case series, pilot studies, retrospective studies, cohort studies, reviews, animal or in vitro studies, and any publications using duplicate data were excluded. Studies using trans-sinus implants and comparing the use of short implants (not associated with the sinus lift technique) with long implants (inserted contextually to the lift) were not considered.

Selection of studies:

Two reviewers (G.G. and L.M.) independently read the titles, the abstracts, and the full texts of the selected articles to ensure they met the inclusion criteria. In cases of disagreement, a third co-author (P.P.) was consulted.

### 2.4. Data Extraction

After the selection of the articles to be included in the review, the data were collected by two authors (L.M. and G.G.) using a Microsoft Excel spreadsheet (Excel 16.4, Microsoft CO., Redmond, WA, USA). The following information was extracted: year and journal of publication, authors, title, type of study (parallel or split-mouth), country in which the study was performed, presence of sponsors, number of operators, presence or absence of smoking patients, total number of patients and maxillary sinuses treated and evaluated, age and sex of patients, total number of implants inserted and type (contextual or delayed insertion with the sinus lift), follow-up period in months, outcomes, grafting materials used, use or not of a covering membrane, number of implants inserted in each group, number of failed implants with relative timing, any post-operative complications found, residual vertical bone dimension prior to surgery, volumetric bone gain, and finally, histomorphometric data on the amount of new bone obtained. The biomaterials used in the various studies were reported based on the category they belong to, i.e., autologous graft (AU), xenograft (XG), allograft (AG), alloplastic material (AP), autologous tissue graft (ATG), biological material (BIO), or for spontaneous healing (only membrane elevation) without the use of any material (SH). 

If the article contained the total linear measurements before and after surgery, the actual bone gain was calculated by subtracting the preoperatively measured value from the post-operative one; this measure was reported in mm ± SD. 

For the volumetric measurement, the volume found at least 6 months after surgery was reported in mm^3^ ± SD along with the percentage of contraction with respect to immediate post-surgery graft volume. 

In cases of missing or unclear data, the authors of the various articles were contacted by e-mail to obtain further details.

### 2.5. Risk of Bias Assessment

The risk of bias (RoB) assessment was conducted according to the Cochrane criteria, *Cochrane Handbook for Systematic Reviews of Interventions* [38]. All the different RoB domains were evaluated for each RCT: random sequence generation (selection bias), allocation concealment (selection bias), blinding of participants and personnel (performance bias), blinding of outcome assessment (detection bias), incomplete outcome data (attrition bias), and selective reporting (reporting bias).

If a potential bias could cause a serious weakness of confidence in the results, the associated risk was defined as high. Otherwise, the risk of bias was classified as “low” if it did not alter the results, or as “unclear” if it raised some doubts about the results of the study.

### 2.6. Statistical Analysis

It was planned to undertake a meta-analysis of the graft shrinkage for each comparison between materials if at least three studies with the same comparison were found. However, no meta-analysis could be performed as there were no more than two studies for each type of comparison. Therefore, no meta-analysis was performed. Linear regression analysis was undertaken between outcome variables (NBF, graft shrinkage percentage) and baseline data (RBH, post-surgery graft height). GraphPad Prism 5.1 (GraphPad, La Jolla, CA, USA) was used as the statistical software. 

## 3. Results

### 3.1. Bibliographic Search and Study Selection

**Figure 1:**
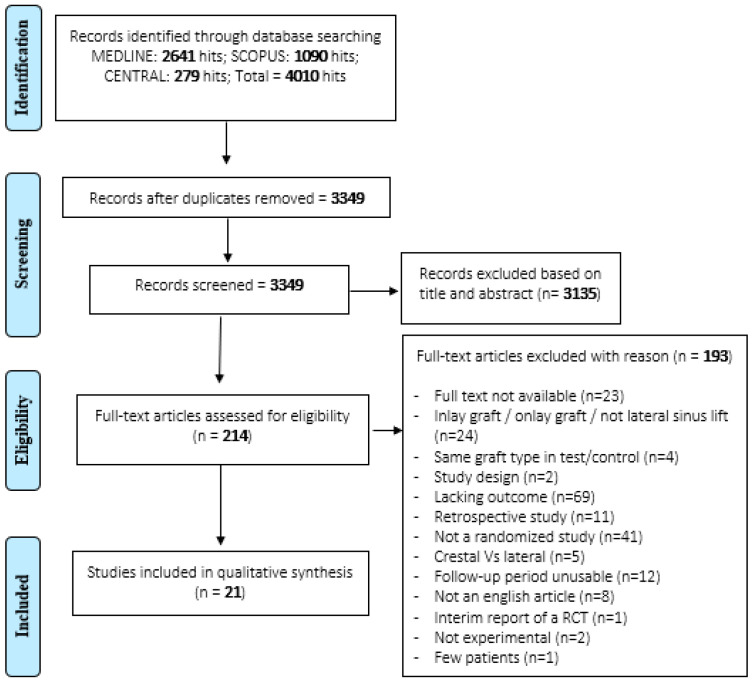
Flowchart of the included studies.

Figure 1 reports the flowchart of the selection process. The electronic search within the three databases produced a total of 4010 articles, with 2641 obtained from PubMed/MEDLINE, 1090 from Scopus, and 279 from the Cochrane Central Register of Controlled Clinical Trials (CENTRAL). After removing 661 duplicates, 3349 articles were analysed. Following the reading of the titles and the abstracts, 3135 were excluded; the remaining 214 were examined using the full text. This research phase excluded 193 articles because they did not comply with the eligibility criteria and made it possible to include a total of 21 publications [39,40,41,42,43,44,45,46,47,48,49,50,51,52,53,54,55,56,57,58,59]. Reasons for exclusion are reported in Supplementary Table S1 and Figure 1.

To obtain some missing information, the authors of 10 articles were contacted [39,41,43,44,45,54,56,57,58,59]. Unfortunately, only three of them replied.

### 3.2. Description of Included Studies

The 21 articles included in the review presented a comparison among at least two different biomaterials: 8 of them were parallel studies [41,45,47,48,50,54,55,57], 11 split-mouths [39,40,42,43,44,49,51,52,56,57,59], and 2 presented both modalities [46,53].

Most of the publications were not sponsored, and only six articles were financed [40,45,46,49,50,54].

In total, 412 patients and 533 sinuses were analysed with 319 for the first PICO question and 301 for the second one. In 12 studies, smokers were excluded from the patient’s selection [39,40,42,43,44,45,46,53,54,55,57,58] while the other 10 articles included smoking patients in the sample. 

Four studies presented a population with a mean age below 50 years [45,49,50], 11 between 50 and 60 years [40,41,43,47,48,52,53,55,57,58,59], and 2 had patients with a mean age of more than 60 years [44,56]. In three studies, the age was reported only as the median value as 55 years [47,51] and 50.5 years [39]. In another two articles, the ranges are included as between 40–77 [46] and 45–62 years [42]. One study did not report this data [54].

In only two studies, the implant insertion was performed simultaneously with the lateral sinus lift procedure [41,48]; in 11 other studies, the implants were inserted at the secondary surgical stage 6 months later [39,43,45,47,49,53,56,57,58,59]. In a study by Panagiotou et al. [52], the fixtures were inserted 8 months after sinus lift; in that of de Lange et al. [44], between 3 and 8 months (average time of 6 months), and in those of Chackartchi et al. [40] and Lorenz et al. [51], between 5 and 9 months. Only the study by Lee et al. [50] did not specify the timing of implant insertion. 

While in most RCTs, the surgical procedure involved the use of a resorbable membrane covering the lateral window, three studies did not consider their use [48,56,59], and two did not specify it [43,54]. Finally, an article described the use of a biological glue in one group, and the same glue was added with autologous PLT concentrates (APCs) in another [39].

Histomorphometrical and vertical height data were reported by 13 studies, including 5 studies that compared different types of heterologous grafts [40,43,50,52,53], 1 that utilized two different homologous bone [41] while 2 studies compared alloplastic materials with each other [45,47]. The remaining RCTs compare, instead, AU + PC against AG + XG + PC [48], AU against AU + PC [39], AU and AP [59], XG and AP [44], and, finally, XG juxtaposed with XG + PC [56]. In general, AG was employed in 34 sinuses, XG in 81, AP in 109, AU in 21, AU + PC in 28, AU + XG + PC in 15, XG + PC in 5, and finally, XG + AP in 6.

Volumetric changes were considered in 11 articles. Three studies compared the use of alloplastic material with autologous bone and/or their combination [49,54] while another 2 studies compared two different materials from the same family including one that utilized alloplastic grafts [47] while the other used heterologous [50]. The remaining RCTs compared: AG with AG + XG [55], AP with XG [51], AU with AG [58], XG with AG [57], AU with XG + AU [42] and, finally, XG + AP with XG [53]. Altogether, AU was used in 49 sinuses, AU + AP in 17, AP in 105, AG in 47, AG + XG in 17, XG in 50, XG + AU in 10, and finally, XG + AP in 6 sinuses.

Nine studies did not specify the number of implants placed after the lateral sinus lift [40,42,45,49,50,52,53,54,56] while one declared to have inserted 2–5 textures in the site without further clarification [39]. One author did not report the insertion of implants [46]. In all other studies, 620 implants were inserted: 55 in sinus grafted with AU, 111 with XG, 160 with AP, 205 with AG, 28 with AU + PC, 31 with AU + XG + PC, and, finally, 30 with AG + XG.

The main characteristics of the included studies are summarized in Supplementary Table S2.

### 3.3. Risk of Bias

**Figure 2:**
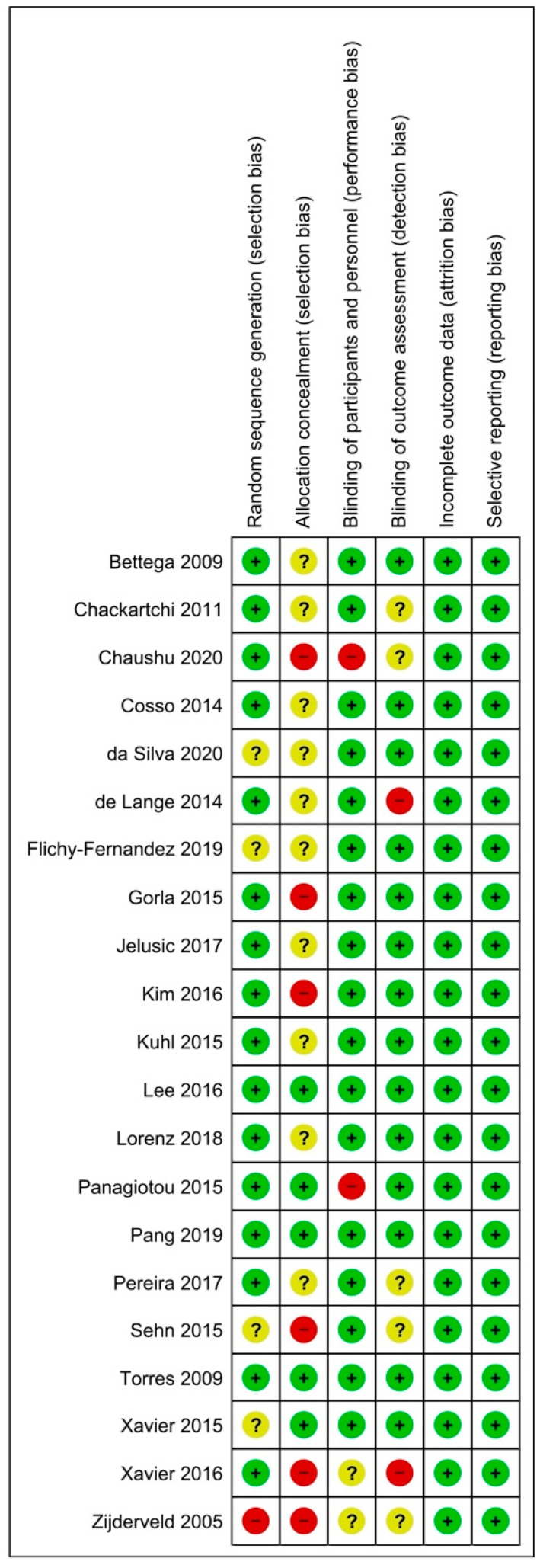
Risk of bias—Green low risk of bias, yellow unclear risk, red high risk of bias.

The evaluation of the risk of bias (RoB) of the included studies (Figure 2) showed that three of them had a low RoB [50,53,56]. Four studies were considered at unclear risk for selection bias–random sequence [43,45,55,58] and one at high risk [59] while, for allocation concealment, ten had an unclear RoB [39,40,42,43,44,45,47,49,51,54] and six, a high risk [41,46,48,55,57,59]. When estimating the risk associated with the blindness of participants and staff (i.e., performance bias), two studies were unclear [57,59], and two with high RoB [41,52] were highlighted. The evaluation of the detection bias revealed the presence of five studies classified as unclear [40,41,54,55,59] and two as high [44,57]. Finally, about attrition and reporting bias, all studies had low risk.

### 3.4. Residual Bone Height and Post-Operative Vertical Change on New Bone Formation

Thirteen studies presenting histomorphometric analyses, that mean residual (pre-operative) bone height (RBH), and the postoperative bone height (PBH) for both test and control groups were included. In 10 studies, the biopsies were taken 6 months after sinus lift [39,43,44,45,47,48,50,53,56,59] while in the remaining three articles, respectively, 9 months [41], 8 months [52], and between 6 and 9 months [40] from the first surgery. 

**Figure 3:**
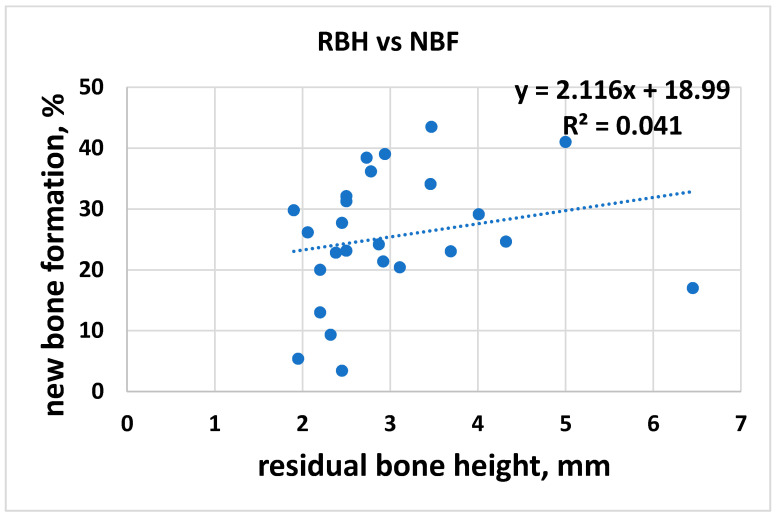
RBH and NBF.

**Figure 4:**
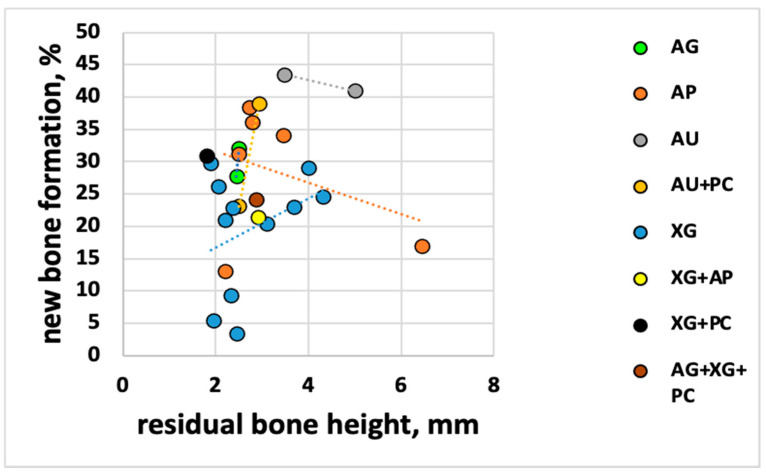
RBH and NBF for each biomaterial.

In the first analysis, all the histomorphometric data of new bone formation percentage (NBF%) values (i.e., mean values) were correlated with the respective mean RBH. The hypothesis was that the NBF% would increase when the RBH increased. The overall linear regression analysis showed a slight agreement with the hypothesis (Figure 3) with a regression coefficient R^2^ = 0.04 and a slope of 2.11 (95% CI = −2.19 to 6.43), meaning that NBF increases by 2.1% per each mm increase in residual bone height; therefore, the NBF was essentially independent of preoperative bone height. In other words, the observed increase in new bone formation with the increasing RBH was not significant (*p* = 0.32). Figure 4 shows that this analysis had different variable trends for each biomaterial (i.e., AG; AP; AU; AU + PC; and XG).

**Figure 5:**
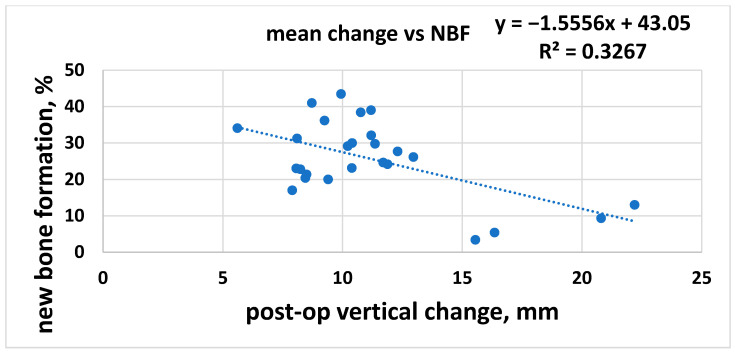
Post-operative vertical change and NBF.

In the second analysis, the NBF% was correlated with the postoperative height (RBH + vertical graft dimension) with the hypothesis that the higher the graft, the lower the new bone formation inside the graft. The overall linear regression analysis showed a tendency in agreement with the hypothesis (Figure 5) with a regression coefficient of R^2^ = 0.33 and a slope of −1.56 (95% CI: −2.50 to −0.61), meaning that NBF significantly decreased when postoperative vertical graft dimension increased. Per each mm of vertical height, NBF changed by about 1.6%. The trend was highly significant (*p* = 0.0023). 

**Figure 6:**
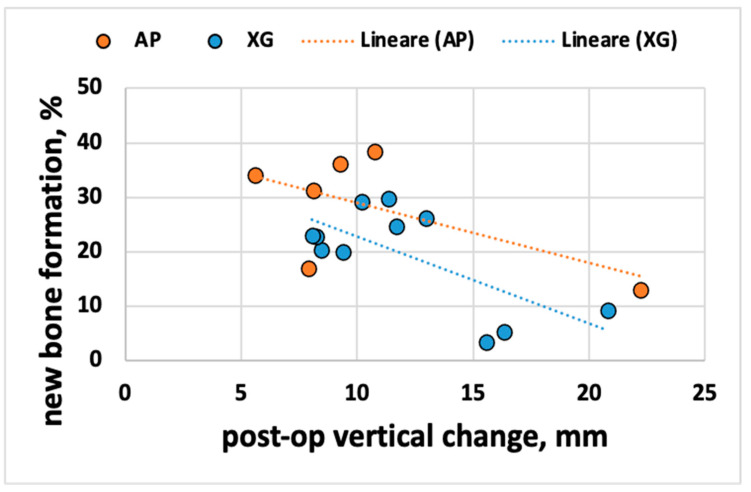
Post-operative vertical change and NBF for each biomaterial.

It seemed interesting to investigate if the correlation was the same or different among the different materials. Since the number of samples per material was limited, a comparison could only be attempted between alloplasts (n = 6) and xenografts (n = 11) (Figure 6). The two linear regressions were almost overlapping, showing a similar tendency, with a regression coefficient even higher than the overall one (R^2^ = 0.37 for alloplasts and R^2^ = 0.48 for xenografts).

The postoperative bone height (PBH), which was correlated to new bone formation, was obtained by adding the RBH and the graft vertical dimension itself. Given that the graft dimension was dependent on the amount of graft inserted by the operator at the surgical time, it was a variable that could be controlled to increase the expected new bone formation, as opposed to the residual bone height.

### 3.5. Shrinkage of Different Bone Substitutes

Regarding graft volume changes, eleven RCTs were considered with a total of 302 sinuses. Graft volume was estimated using CBCT, and volume change was calculated by subtracting the value at the 6-month follow-up from the baseline value. Data were grouped by grafting material. Five groups were considered including autogenous graft (AU, n = 4), xenograft (XG, n = 5), alloplast (AP, n = 6), allograft (AG, n = 3), and autogenous + alloplast (AU + AP, n = 2). The data of volume reduction in mm^3^ were normalized to the percentage of the reduction and dividing by the baseline values. Other grafts composed by combined materials (AG + XG, XG + AP, and XG + AU) were not considered for comparison because each of them was investigated by a single study.

**Figure 7:**
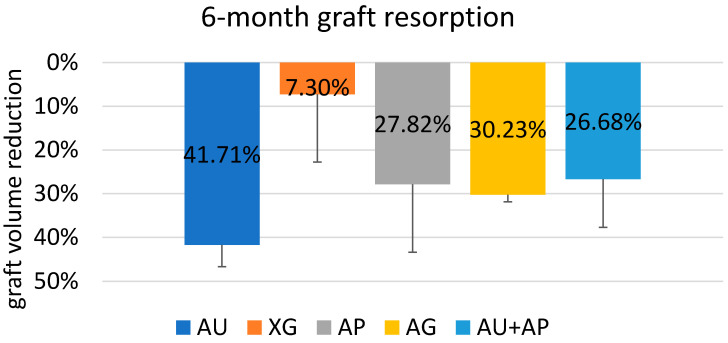
Percentage reduction for each biomaterial.

After 6 months, the material showing the lowest volume reduction was XG (7.30 ± 15.49%) while the one that resorbed most was AU (41.71 ± 12.63%). For the other biomaterials, a volumetric contraction of 27.82 ± 15.58% was evaluated for the AP, 30.23 ± 1.61 for the AG, and finally, 26.68 ± 11.03% for AU + AP (Figure 7). Significant differences were only found between AU and XG (*p* = 0.009) and between AG and XG (*p* = 0.048) (Supplementary Table S3). An unpaired *t*-test was used for comparison. Other comparisons did not show significant differences.

### 3.6. Implant Survival

From the analysis of the data extrapolated from the articles, only 10 implants, among 620 inserted, failed: three of those were placed in sinuses grafted with AG [55,57], three in sinus with AP [44,47,51], and three with XG [43,51]. The RCT of Flichy-Fernández et al. [45] reported the failure of one implant without, however, specifying in which group it occurred, and so it wasn’t considered (Supplementary Tables S4 and S5).

#### Post-Operative Complications

Post-operative complications were not reported in five articles [42,45,46,51,56]. In nine studies, no unexpected events were observed, both in the surgical phases and in the postoperative course [39,41,44,48,53,54,55,57,58]. 

Da Silva et al. [43] found four intraoperative perforations of the Schneiderian membrane with one of them occurring in a site grafted with Bio-Oss while the remaining three in the group were treated with Lumina-Bone Porous. The study by Jelusic et al. [47] detected the highest number of postoperative complications including five membrane perforations in the group treated with β-TCP and eight in the one treated with an alloplastic graft composed of 60% HA and 40% β-TCP. Lee et al. [50], in addition to a perforation of the Schneiderian membrane of 10 mm in diameter that occurred in the group treated with DPBM, reported the appearance of extensive but temporary postoperative swelling in a site treated with DBBM, mainly due to patient negligence. Panagiotou et al. [52] reported the perforation of the Schneiderian membrane in two sites among those treated with Bio-Oss, while de Lange et al. found the same intraoperative complication in four of the sites grafted with AU and in two of those treated with AP [44]. Kuhl et al. [49] detected two perforations, one in the AP group and one in AP + AU.

In the split-mouth study by Chackartchi et al. [40], a patient had a bilateral postoperative infection involving both sinuses in comparison, respectively treated with XG having small (0.25–1 mm) and large (1–2 mm) size particles. In the RCT by Pereira et al. [54], a similar situation affected one patient belonging to the AP + AU group. These complications were resolved by administering antibiotic therapy. 

## 4. Discussion

The aim of the present systematic review based on RCTs had been to investigate if there was an effect of residual bone height (RBH) and vertical bone gain on new bone formation (NBF) as well as the shrinkage of the graft when using different biomaterials. 

Graft volumetric shrinkage occurring after SLs using different biomaterials [46,54,60,61] influenced implant placement in two-stage lateral sinus lifts and could impact implant survival over time [62]. Furthermore, while complete resorption and replacement of the graft by a new bone formation is desirable, in some cases, a long-lasting scaffold was crucial to support osteointegrated implants [59]. Regarding the AU reabsorption, a recent meta-analysis by Starch-Jensen et al. [63] and a systematic review by Shanbhag et al. [64] confirmed our results. Unfortunately, in the present work, no meta-analysis of graft shrinkage could be performed as there were no more than two studies for each type of comparison between materials. Xenografts show a very slow resorption pattern [26,28,29]. This agrees with data obtained from this review and with those reported by Mazzocco et al. (7%, at 8 months, ranging from −29% to 18%, for the delayed implant placement subgroup; and 13%, at 8 months, ranging from −24% to −3%, for the simultaneous implant placement subgroup) [65], and by Gutekin et al. (8.14 ± 3.76%, at 6 months) [66]. Higher resorption values were instead reported after 6 months using deproteinized bovine bone by Salem et al. in a prospective randomized clinical pilot study (23.8% ± 15.9%) [67] and by Zhang et al. in a cohort study (22.7%) [62].

The resorption rate of AP, AG, and AU + AP indicated that greater graft stability may be achieved using bone substitutes or a composite graft, which then added AP to the patient’s bone, compared to using AU alone. Similar conclusions were drawn by Shanbhag et al. in their review, which further stipulated that neither two-stage implant placements nor survival rates of simultaneously placed implants were affected by graft volume reductions [64]. 

Some authors suggested that the amount of new bone formation (%) was a reliable parameter of bone graft performance since the greater that the quality of the newly formed bone was, the greater the BIC (bone-implant contact) will be, and, eventually, the implant survival [35]. Several reviews agreed, considering AU the gold standard in terms of new bone formation [21,34,35]. In particular, Al-Moraissi et al. recommended the use of only AU if the implant rehabilitation was planned within 6 months [21]; however, while Handschel et al. agreed, they suggested a similar course if the functional loading was expected within 9 months of sinus lift [68]. 

The NBF, as well as the material used in the sinus lift, may also be affected by sinus morphology and dimension; reduced osteogenic potential occurs in larger sinus cavities where more time is required to obtain adequate bone formation because of the greater distance between the sinus walls and the augmented region [69,70]. In narrow sinuses, it is more likely to achieve a better sinus membrane reflection as well as intimate contact between grafting material and sinus walls during transcrestal sinus floor elevation [70]. Considering that bone formation arises from the peripheral bony walls toward the centre of the graft [70], as we have seen in post-extraction socket healing, delayed or scarce graft maturation could occur if the preoperative amount of residual bone is minimal. Similarly, Corbella et al. hypothesized that if the sinus floor had only a thin layer of cortical bone, a lower percentage of NBF was to be expected, as compared to less atrophic cases in which the additional presence of cancellous bone could guarantee better regeneration potential [34]. Since the regenerative potential starts from the residual bone, NBF may also be affected by the harvesting technique and by the vertical gradient of graft consolidation in the collected specimens [71]. Beck et al., analysing histological thin-ground sections, demonstrated that NBF decreased with increasing distance from the residual sinus floor since the residual graft encapsulated by fibrous tissue had remained in the apical part of the augmented area [71].

Our results, revealing that residual bone height was not a remarkable factor affecting graft maturation, are in accordance with those reported by previous histomorphometric studies analysing biopsies retrieved 6 months after lateral sinus lift had been performed with XG [72] and AP [73]. Pignaton et al. [74] also confirmed that both RBH and sinus width were not influencing factors on new bone formation 8 months after lateral sinus lift with anorganic bovine bone.

Kühl et al. considered overaugmentation preferential in two-stage sinus lifts to compensate for expected graft volumetric reduction at the time of implant placement [75]. On the contrary, our results seemed to suggest that excessive vertical augmentations immediately after sinus floor elevation could lead to lower NBF at the time of the second surgery, even if a shrinkage of the material was always present. 

Regarding the correlation between PBH and NBF, further studies are needed to investigate these hypotheses further and to understand the clinical significance of reducing the graft size in order to increase the NBF.

It has long been shown that NBF occurs in the maxillary sinus via sinus mucosa lifting without using any graft type [76,77,78], supporting the thesis of the osteogenic capability of the Schneiderian membrane. Although the debate is ongoing, this hypothesis has been confirmed by in vivo and in vitro studies [79,80] that also demonstrated the presence of mesenchymal progenitor cells and cells committed to the osteogenic lineage in the membrane itself [81,82]. The obtained results suggested that a lower Schneiderian membrane elevation may be sufficient to trigger its osteogenic potential efficiently, as compared to grafting a greater amount of biomaterial, which would be counterproductive. Similarly, Beck et al. also concluded that a large augmentation height, particularly in the molar region, would not promote high NBF in an apical area within the grafted region [71].

One of the limits of the present review is the great variability of sinus morphology as sinus dimensions can influence the percentage of new bone formation, as has been shown by Stacchi et al. [83].

In addition, these conclusions could not be confirmed or denied as the current literature lacks RCTs, reviews, and meta-analyses assessing the correlation between vertical bone gain and histomorphometric outcomes. 

## 5. Conclusions

Within the limits of the present systematic review, the following conclusions could be drawn:

Six months after lateral sinus lift, the material showing the lower volume reduction was the XG while the one that resorbed most was the AU, according to the current literature. 

Since the analysis of the correlation between NBF and RBH had revealed a non-significant increase in new bone formation when residual bone height increased, NBF is essentially independent of preoperative bone height.

NBF significantly decreased when postoperative vertical graft dimension increased, supporting the hypothesis that the higher the graft height, the lower the new bone formation within it. Therefore, at surgical time, the operators should carefully control the amount of grafted biomaterial applied to optimize the expected NBF.

## Data Availability

Data available on request.

## Supplementary Materials

The following are available online at https://www.mdpi.com/article/10.3390/jcm10214996/s1, Table S1: List of the excluded studies with the main reason for exclusion, Table S2: Main characteristics of the included studies, Table S3: P-values of the comparisons among groups, Table S4: Main characteristics of the included studies for volumetric changes, Table S5: Main characteristics of the included studies for histomorphometric and linear analisys.
