# Supplementary material for: Radiographic and Histomorphometric Evaluation of Biomaterials Used for Lateral Sinus Augmentation: A Systematic Review on the Effect of Residual Bone Height and Vertical Graft Size on New Bone Formation and Graft Shrinkage
Source: J Clin Med. 2021 Oct 27;10(21):4996. doi: 10.3390/jcm10214996 (PMC8584826; doi:10.3390/jcm10214996)
Supplement: Supplementary file 1 [file jcm-10-04996-s001.zip › jcm-1415471-supplementary.pdf]

SUPPLEMENTARY TABLES

Table S1. List of the excluded studies with the main reason for exclusion.

Authors	Reason for exclusion
Adileh Shirmohammadi et al. 2014	Follow-up period unusable
Aleksa Markovic et al. 2016	Inlay graft / onlay graft / not lateral sinus lift
Amir Dasmah et al. 2013	Inlay graft / onlay graft / not lateral sinus lift
Andreas Thor et al. 2005	Inlay graft / onlay graft / not lateral sinus lift
Angelo Troedhan et al. 2014	Inlay graft / onlay graft / not lateral sinus lift
Antonio Barone 2005	Follow-up period unusable
Antonio Barone et al. 2006	Not a randomized study
Antonio Scarano et al. 2006	Not a randomized study
Arne Mordenfeld et al. 2014	Not a randomized study
Arne Mordenfeld et al. 2016	Lacking Outcome
Arne Mordenfeld et al.2014	Inlay graft / onlay graft / not lateral sinus lift
Atsuya Hirota et al. 2019	Lacking Outcome
B Johansson 1999	Inlay graft / onlay graft / not lateral sinus lift
B Johansson et al. 1999	Lacking Outcome
Babbush CA. 1998	Full-text not available
Bjarni E Pjetursson et al. 2009	Not a randomized study
Bjarni E. Pjetursson 2009	Not a randomized study
Block MS 1993	Retrospective study
Blomqvist JE. 1998	Full-text not available
Bori J. 1993	Full-text not available
Boskovic MM 1990	Full-text not available
C Karabuda et al. 2001	Not a randomized study
C Maiorana 2005	Not a randomized study
C Mangano 2007	Lacking Outcome
C Y S Lee 2008	Lacking Outcome
Cameron Y S Lee et al. 2011	Not a randomized study
Cannizzaro G et al. 2013	Crestal Vs Lateral
Carolina Sbordone et al. 2013	Retrospective study
Ceyda Kanli Dursun 2016	Lacking Outcome
Cheng Y. et al. 2017	Not an english article
Christer Lindgren et al. 2012	Lacking Outcome
Christer Lindgren et al. 2012	Lacking Outcome
Christine Knabe et al. 2017	Lacking Outcome
Christoph Zizelmann et al. 2007	Follow-up period unusable
Cravatta CM et al. 2004	Full-text not available
D De Leonardis et al. 2000	Not a randomized study
D Rickert et al. 2011	Follow-up period unusable
D Rickert et al. 2014	Lacking Outcome
Damir Jelušić et al. 2014	Lacking Outcome
Danilo Alessio Di Stefano et al. 2015	Lacking Outcome
Darnell Kaigler 2015	Follow-up period unusable
Deppe H et al. 2012	Not a randomized study
Dong-Seok Sohn et al. 2008	Not a randomized study
Elton Carlos Pichotano et al. 2019	Follow-up period unusable
Engelbert A.J.M. Schulten et al. 2013	Not a randomized study
Eun-Sik Kim et al. 2013	Crestal Vs Lateral
F Cabbar 2011	Lacking Outcome
F Inchingolo et al. 2012	Not a randomized study
F L Borges 2011	Lacking Outcome
Fabrício Moreira Serra et al. 2006	Lacking Outcome
Fawzi Riachi et al. 2012	Lacking Outcome

Fernando Verdugo et al. 2017	Inlay graft / onlay graft / not lateral sinus lift
Florin Onişor-Gligor et al. 2015	Not a randomized study
Fu Z-F et al. 2015	Full-text not available
G Corinaldesi 2013	Follow-up period unusable
G La Monaca 2018	Lacking Outcome
G M Raghoebar 2001	Retrospective study
G M Raghoebar et al. 1997	Not a randomized study
G Szabó et al. 1999	Not an english article
G Tawil 2001	Not a randomized study
G. M. Raghoebar 1999	Not a randomized study
G. Watzek 1998	Retrospective study
Garg AK 2001	Full-text not available
Garg AK 2008	Full-text not available
Gurumoorthy Kaarthikeyan et al. 2019	Lacking Outcome
György Szabó et al. 2005	Lacking Outcome
H Ozyuvaci et al. 2003	Not a randomized study
Hai Anh Trinh et al. 2019	Inlay graft / onlay graft / not lateral sinus lift
Hallman M et al. 2002	Not an english article
Heidrun Schaaf et al. 2008	Lacking Outcome
Henk-Jan Prins et al. 2016	Not a randomized study
Hong-Chang Lai et al. 2010	Inlay graft / onlay graft / not lateral sinus lift
Hossam Khaled et al. 2019	Lacking Outcome
Huajie Yu et al. 2017	Same graft type in test/control
J H Lee 2008	Not a randomized study
J I Smedberg 2001	Inlay graft / onlay graft / not lateral sinus lift
J S Cavallaro 2010	Not experimental
J W Olson 1997	Interim report of a RCT
J W Olson et al. 2000	Not a randomized study
Jamil Alayan et al. 2018	Not a randomized study
Jamil Alayan et al. 2019	Not a randomized study
Ji-Su Oh 2019	Lacking Outcome
John C Minichetti et al. 2008	Not a randomized study
Josh Whitt et al. 2020	Follow-up period unusable
JT García-Denche 2013	Lacking Outcome
Juliana Dreyer Menezes et al. 2018	Lacking Outcome
Justin Ranaan et al. 2018	Lacking Outcome
KE Kahnberg 2001	Inlay graft / onlay graft / not lateral sinus lift
KE Kahnberg 2008	Not a randomized study
Kelston Ulbricht Gomes et al. 2008	Inlay graft / onlay graft / not lateral sinus lift
Khalid Bahaa-Eldin et al. 2017	Few patients
Kyung-In Jeong et al. 2011	Lacking Outcome
Laith A Hussein et al. 2017	Inlay graft / onlay graft / not lateral sinus lift
Lars Rasmusson et al. 2012	Lacking Outcome
Lars-Ake Johansson et al. 2010	Not a randomized study
Lars-Ake Johansson et al. 2013	Lacking Outcome
Leonardo Trombelli et al. 2012	Inlay graft / onlay graft / not lateral sinus lift
Leonardo Trombelli et al. 2014	Inlay graft / onlay graft / not lateral sinus lift
Lozada JL et al. 1993	Full-text not available
M B Hürzeler et al. 1996	Lacking Outcome
M Badr 2010	Not lateral sinus lift
M Esposito 2010	Lacking Outcome
M M Bornstein 2008	Not a randomized study
M S Block et al. 1998	Retrospective study
M Tatullo 2012	Lacking Outcome
M Tosta 2013	Lacking Outcome
Marcelo Carlos Bortoluzzi et al. 2014	Same graft type in test/control
Marco Portelli et al. 2017	Lacking Outcome
Marius Steigmann et al. 2005	Crestal Vs Lateral

Marta Krasny et al. 2013	Not a randomized study
Massimo Del Fabbro et al. 2015	Lacking Outcome
Mats Hallman et al. 2002	Not a randomized study
Mats Hallman et al. 2002	Lacking Outcome
Maurizio Silvestri 2013	Lacking Outcome
Mauro Merli et al. 2013	Lacking Outcome
Mazor Z et al. 2000	Full-text not available
Metzler C 2000	Full-text not available
Mi-si Si et al. 2013	Inlay graft / onlay graft / not lateral sinus lift
Michael S Block et al. 2006	Not experimental
Michele Cassetta et al. 2015	Lacking Outcome
Minsk L. 2003	Full-text not available
Namineni Kiran Kumar et al. 2015	Not a randomized study
Navarro I. 1998	Full-text not available
Nevins M. 1995	Full-text not available
Niels Ulrich Hermund et al. 2012	Follow-up period unusable
Norbert Velich et al. 2004	Not a randomized study
Nuray Yilmaz Altintas et al. 2013	Lacking Outcome
Nynke Lie et al. 2015	Lacking Outcome
Ofer Mardinger et al. 2011	Retrospective study
P Felice 2009	Follow-up period unusable
P Galindo-Moreno 2010	Not lateral sinus lift
P J Boyne 2005	Follow-up period unusable
Pablo Galindo-Moreno et al. 2008	Not a randomized study
Palti A. 2003	Full-text not available
Paul A Fugazzotto et al. 2007	Retrospective study
Peter Rammelsberg et al. 2020	Not a randomized study
Pit Voss et al. 2010	Not a randomized study
R. Gilbert Triplett 1996	Retrospective study
R. Gilbert Triplett 2009	Lacking Outcome
Rabah Nedir et al. 2013	Inlay graft / onlay graft / not lateral sinus lift
Rabah Nedir et al. 2016	Inlay graft / onlay graft / not lateral sinus lift
Rabah Nedir et al. 2017	Inlay graft / onlay graft / not lateral sinus lift
Rabah Nedir et al. 2019	Inlay graft / onlay graft / not lateral sinus lift
Ran Herzberg et al. 2006	Retrospective study
Reiche O et al. 1991	Full-text not available
Roberto Crespi et al. 2007	Follow-up period unusable
S F Belouka 2016	Lacking Outcome
S M Meloni 2015	Lacking Outcome
S S Noumbissi 2005	Not a randomized study
S V Sirak et al. 2016	Not an english article
Sameh Attia et al. 2020	Retrospective study
Samer Kasabah 2002	Not a randomized study
Samuel P. Xavier 2015	Lacking Outcome
Sean P. Avera 1997	Lacking Outcome
Sebastian Kühl et al. 2012	Lacking Outcome
Sebastian Sauerbier et al. 2010	Lacking Outcome
Selen Adiloglu et al. 2019	Lacking Outcome
Sergio Di Lallo et al. 2014	Lacking Outcome
Shin Ogawa et al. 2016	Inlay graft / onlay graft / not lateral sinus lift
Silvio Mario Meloni et al. 2017	Lacking Outcome
Silvio Mario Meloni et al. 2019	Lacking Outcome
Silvio Taschieri et al. 2016	Not a randomized study
Simons AM. 1992	Full-text not available
Stephen S Wallace et al. 2005	Same graft type in test/control
Stephens WL. 1994	Full-text not available
Steven A Zijderveld et al. 2009	Lacking Outcome
Stuart J Froum 1998	Crestal Vs Lateral

Suba Z et al. 2004	Not an english article
Suba Z et al. 2006	Not an english article
Summers RB. 1996	Full-text not available
Summers RB. 1996	Full-text not available
T.L.M.R. Dos Anjos 2016	Lacking Outcome
Thomas Zumstein et al. 2016	Lacking Outcome
Tim Fienitz et al. 2016	Lacking Outcome
Tomasetti B. 2004	Full-text not available
Triplett RG et al. 1994	Full-text not available
Ugo Consolo 2007	Not a randomized study
Valérie Diserens et al. 2005	Inlay graft / onlay graft / not lateral sinus lift
Velich N et al. 2003	Not an english article
Volker Gassling et al. 2013	Same graft type in test/control
Waleed Fouad et al. 2018	Lacking Outcome
Wang Peng et al. 2013	Lacking Outcome
Wataru Katagiri et al. 2017	Not a randomized study
Wilfried Wagner et al. 2012	Lacking Outcome
Winnie Pradel et al. 2008	Not a randomized study
Wohlfahrt C.2003	Full-text not available
Xu Zhao et al. 2018	Study design
Xu Zhao et al. 2018	Study design
Young-Kyun Kim 2009	Retrospective study
Young-Kyun Kim et al. 2014	Crestal Vs Lateral
Zahid Z.M. et al. 2015	Lacking Outcome
Zhang X et al. 2012	Not an english article
Zvi Schwartz et al. 2007	Not a randomized study

Table S2. Main characteristics of the included studies.

Authors	Year	Sponsor	Parallel (P) / split mouth (SM)	Smokers incl. Y/N	Patients / sinus evaluated	Age and M/F	Contestual sinus and implant insertion Y/N	Material 1	Material 1 Cover with or without membrane	N. implant material 1	Material 2	Material 2 Cover with or without membrane	N. implant material 2	Material 3	Material 3 Cover with or without membrane	N. implant material 3
Gorla et al.	2015	Y	P + SM	N	20 / 32	40 - 77 yrs; 16F, 6M	N	AU	without	0	AU + AP	without	0	AP	without	0
Sehn et al.	2015	N	P	N	29 / 34	51.32 ± 6,44 yrs; 21 F, 8M	N	AG	collagen membrane	40	AG + XG	collagen membrane	30			
Lorenz et al.	2018	NR	SM	Y	14 / 28	55 yrs; 5M, 9F	N	AP	collagen membrane	26	XG	collagen membrane	27			
Xavier et al.	2015	N	SM	N	15 / 30	54±5 yrs; 8M, 7F	N	AU	collagen membrane	40	AG	collagen membrane	40			

Kühl et al.	2015	Y	SM	Y	8 / 16	7 F: 47.7 yrs ±11.7; 1 M: 58 yrs	N	AP	collagen membrane	NR	AP + AU	collagen membrane	NR			
Xavier et al.	2016	N	P	N	30 / 30	51.17 ± 10.86 yrs; 11M, 19F	N	XG	collagen membrane	35	AG	collagen membrane	35			
Cosso et al.	2014	N	SM	N	10 / 20	45-62 yrs; 4M, 6F	NR	AU	without	NR	XG + AU	without	NR			
Pereira et al.	2018	Y	P	N	27 /33	NR	N	AU	NR	NR	AP	NR	NR	AP + AU	NR	NR
Jelusic et al.	2017	N	P	Y	60 / 60	55.92 yrs; 32M, 28F	N	AP	collagen membrane	47	AP	collagen membrane	45			
Lee et al.	2016	Y	P	Y	11 / 15	M1: 49.17 ±1.33 yrs;	N	XG	collagen membrane	NR	XG	collagen membrane	NR			

						M2: 40.83 ± 9.97 yrs; 12M, 3F										
Pang et al.	2019	N	P + 3 SM	N	12/ 12	M1: 56.67 ± 10.53 yrs. M2: 52.89 ± 12.69 yrs. 14M, 14F	N	XG + AP	with collagen membrane	NR	XG	with collagen membrane	NR			
Chaushu et al.	2020	N	P	Y	29 / 34	55.5 ± 10, yrs; 12M, 17F	Y	AG	With collagen membrane	51	AG	With collagen membrane	39			
da Silva et al.	2020	N	SM	N	13 / 26	55.0 ± 8.13 yrs;	N	XG	NR	15	XG	NR	18			

						6M, 7F										
Kim et al.	2016	N	P	Y	30 / 30	54.6 yrs; 19M, 11F	Y	AU + PC	Without	28	AG + XG + PC	Without	31			
Panagiotou et al.	2015	NR	SM	Y	8 / 16	51.5 ± 10.5 yrs; 4M, 4F	N	XG	With collagen membrane	NR	XG	With collagen membrane	NR			
Bettega et al.	2009	N	SM	N	18 / 36	50.5 (median age); 5M, 13F	N	AU	With biologic glue	From 2 to 5 implant per side	AU + PC	With APCs + biologic glue	From 2 to 5 implant per side			
Zijderveld et al.	2005	N	SM	Y	10 / 16	52.2 yrs; 6M, 4F	N	AU	Without	15	AP	Without	26			
de Lange et al.	2014	N	SM	N	5 / 10	66 yrs; 1M, 4F	N	XG	With collagen membrane	16	AP	With collagen membrane	16			

Flichy-Fernández et al.	2019	Y	P	N	36 / 36	43.11 ± 9.32 yrs; 22M, 14F	N	AP	with resorbable membrane	NR	AP	with resorbable membrane	NR			
Torres et al.	2009	N	SM	NR	5 / 10	61.2 yrs; 2M, 3F	N	XG	Without	NR	XG + PC	Without	NR			
Chackartchi et al.	2011	Y	SM	N	9 / 18	54.25 yrs; 6M, 4F	N	XG	With collagen membrane	NR	XG	With collagen membrane	NR			

Table S3. P-values of the comparisons among groups.

**AU**	**XG**	**AP**	**AG**	**AU+AP**	
	0.009	0.1774	0.1874	0.2296	AU
		0.0571	0.048	0.176	XG
			0.8032	0.9285	AP
				0.5912	AG
					AU+AP

Table S4. Main characteristics of the included studies for volumetric changes.

Authors	N. implant failed material 1	N. implant failed material 2	N. implant failed material 3	Complications material 1	Complications material 2	Complications material 3	Material 1 mean vertical bone T1 (1-2 weeks post-op)	Material 1 mean vert change mm T2 (6-7 months post-op)	Material 1 follow-up Histo months	Material 1 mean new bone %	Material 2 mean vertical bone T1 (1-2 weeks post-op)	Material 2 mean vert change mm T2 (6-7 months post-op)	Material 2 follow-up Histo months	Material 2 mean new bone %	Material 3 mean vertical bone T1 (1-2 weeks post-op)	Material 3 mean vert change mm T2 (6-7 months post-op)	Material 3 follow-up Histo months	Material 3 mean new bone %
Gorla et al.				NR	NR	NR	1.07 cm3 +/- 0.478	0.528 cm3 +/- 0.221			1.295 cm3 +/- 0.937	0.849 cm3 +/- 0.801			0.980 cm3 +/- 0.502	0.56 cm3 +/- 0.336		
Sehn et al.	3 for non-osseointegration	0		N	N		2.75 cm3 +/- 0.73	1.87 cm3 +/- 0.58	6	11.94 +/- 1.71	2.88 cm3 +/- 0.98	2.60 cm3 +/- 0.97	6	25.79 +/- 8.76				
Lorenz et al.	1	1		NR	NR		2.953 +/- 1.249 cm3	2.112 +/- 1.000 cm3	7	19.02 +/- 7.28	3.037 cm3 +/- 0.859	2.358 cm3 +/- 0.76	7	23.66 +/- 7.97				

Xavier et al.	NR	NR		N	N		2.01 cm3 +/- 0.43	1.53cm^3 +/- 0.49	NR	NR	2.46 cm3 +/- 0.79	1.75 cm^3 +/- 0.64	NR	NR				
Kühl et al.	NR	NR		1 perforation	1 perforation		2.34 cm3 +/- 0.38	1.91 cm3 +/- 0.41			2.86 cm3 +/- 0.77	2.32 cm3 +/- 0.57						
Xavier et al.	0	1		N	N		2.9 cm3 +/- 900	2.56 cm3 +/- 800	6	NR	2.48 cm3 +/- 720	1.74 cm3 +/- 820	6	NR				
Cosso e t al.	NR	NR		NR	NR		2.91 cm^3 +/- 1.0	1.7cm^3 +/- 0.9	NR	NR	2.3cm^3 +/-0.9	1.7cm^3 +/- 0.6	NR	NR				
Pereira et al.	NR	NR	NR	N	N	1 patients maxillary contracted sinus infection	1,071 cm3 +/- 0.478	0.528cm3 +/- 0.221			0.909 cm3 +/- 0.472	0.469 cm3 +/- 0.228			1.591 cm3 +/- 0.874	1.006 cm3 +/- 0.589		
Jelusic et al.	1	0		5 perforations	8 perforations		1.73 cm^3 +/- 0.55	1.34 cm^3 +/- 0.45	6	36.16 +/- 19.37	2.0 cm^3 +/- 0.61	1.87 cm^3 +/- 0.6	6	38.42 +/- 12.61				

Lee et al.	NR	NR		1 site extensive but transient facial swelling due to the patient's carelessness	1 perforation		1.74 cm3+/- 0.47	1.90 cm3+/- 0.52	6	26.15 +/- 7.11	1.56 cm3+/- 0.45	1.70 cm3+/- 610	6	29.77 +/- 9.38				
Pang et al.	NR	NR		N	N		1.709 cm3 +/- 0.302	1.324 mm3 +/- 0.302	6	21.37 +/- 8.87	1.910 cm3 +/- 0.945	1.517 cm3 +/- 0.684	6	23.02 +/- 5.88				

Table S5. Main characteristics of the included studies for histomorphometric and linear analisys.

Authors	N. implant failed material 1	N. implant failed material 2	Complications material 1	Complications material 2	Material 1 RBH ± SD (mm)	Material 1 mean vertical change postop ± SD (mm)	Material 1 follow-up Histo (months)	Material 1 mean NBF ± SD (%)	N° Material 1	Material 2 RBH ± SD (mm)	Material 2 mean vertical change postop ± SD (mm)	Material 2 follow-up Histo (months)	Material 2 mean NBF ± SD (%)	N° Material 2
Chaushu et al.	0	0	No	No	2.5 ± 1	11.2 ± 1	9	32.1 ± 19	17	2.45 ± 1	12.3 ± 1	9	27.7 ± 15	17
da Silva et al.	0	2	1 membrane perforation	3 membrane perforations	3.11 ± 0.83	8.45 ± 2.03	6	20.4 ± 5.4	13	2.38 ± 0.75	8.24 ± 1.93	6	22.8 ± 8.5	13
Jelusic et al.	1	0	5 membrane perforations	8 membrane perforations	2.78 ± 1.31	9.25 ± 1.69	6	36.16 ± 19.37	30	2.73 ± 1.06	10.76 ± 1.9	6	38.42 ± 12.61	30
Lee et al.	NR	NR	1 transient facial swelling	1 membrane perforation	2.06 ± 0.43	12.96 ± 3.17	6	26.15 ± 7.11	7	1.9 ± 0.80	11.36 ± 1.89	6	29.77 ± 9.38	8
Kim et al.	0	0	No	No	2.50 ± 1.01	10.39 ± 1.07	6	23.13 ± 1.42	10	2.87 ± 0.74	11.88 ± 1.31	6	24.18 ± 2.19	10
Panagiotou et al.	NR	NR	No	2 membrane perforations	4.01 ± 1.86	10.78 ± 2.02	8	29.13 ± 13.81	29	4.32 ± 1.58	11.84 ± 1.52	8	24.63 ± 19.76	8
Bettega et al.	0	0	No	No	3.47 ± 2.21	9.94 ± 2.67	6	43.48 ± 13.00	12	2.94 ± 1.76	11.19 ± 4.31	6	39.02 ± 10.14	12

Zijderveld et al.	0	0	4 membrane perforations	2 membrane perforations	5 ± 2.05	8.72 ± 2.05	6	41 ± 10	6	6.45 ± 1.67	7.9 ± 1.67	6	17 ± 5	10
de Lange et al.	0	1	No	No	2.32 ± 1.01	20.8 ± 3.77	6 (3 to 8)	9.33 ± 4.73 (mm3)	4	2.2 ± 0.82	22.2 ± 3.63	6 (3 to 8)	13 ± 3.6 (mm3)	4
Flichy-Fernández et al.	1; non reported in which group	1; non reported in which group	NR	NR	2.50 ± 1.58	8.1 ± 4.81	6	31.25 ± 13.82	16	3.46 ± 0.87	5.61 ± 3	6	34.09 ± 14.11	20
Torres et al.	NR	NR	NR	NR	2.2 ± 0.8	9.4 ± 0.7	6	21 ± 5	5	1.8 ± 0.84	10.4 ± 0.7	6	31 ± 5	5
Chackartchi et al.	NR	NR	1 post-op infection (same patient)	1 post-op infection (same patient)	1.95 ± 1.06	16.35 ± 1.12	6 to 9	5.39 ± 4.72 (mm3)	9	2.45 ± 1.46	15.55 ± 2.15	6 to 9	3.41 ± 2.38 (mm3)	9
Pang et al.	NR	NR	No	No	2.92 ± 2.17	8.5 ± 6.07	6	21.37 ± 8.87	10	3.69 ± 4.85	8.07 ± 4.48	6	23.02 ± 5.88	14
